# Modeling of the Bacterial Mechanism of Methicillin-Resistance by a Systems Biology Approach

**DOI:** 10.1371/journal.pone.0006226

**Published:** 2009-07-13

**Authors:** Ida Autiero, Susan Costantini, Giovanni Colonna

**Affiliations:** 1 CRISCEB (Interdepartmental Research Center for Computational and Biotechnological Sciences), Second University of Naples, Naples, Italy; 2 CROM (Oncology Research Centre of Mercogliano) “Fiorentino Lo Vuolo”, Mercogliano, Italy; 3 Department of Biochemistry and Biophysics, Second University of Naples, Naples, Italy; Tata Institute of Fundamental Research, India

## Abstract

**Background:**

A microorganism is a complex biological system able to preserve its functional features against external perturbations and the ability of the living systems to oppose to these external perturbations is defined “robustness”. The antibiotic resistance, developed by different bacteria strains, is a clear example of robustness and of ability of the bacterial system to acquire a particular functional behaviour in response to environmental changes. In this work we have modeled the whole mechanism essential to the methicillin-resistance through a systems biology approach. The methicillin is a β-lactamic antibiotic that act by inhibiting the penicillin-binding proteins (PBPs). These PBPs are involved in the synthesis of peptidoglycans, essential mesh-like polymers that surround cellular enzymes and are crucial for the bacterium survival.

**Methodology:**

The network of genes, mRNA, proteins and metabolites was created using CellDesigner program and the data of molecular interactions are stored in Systems Biology Markup Language (SBML). To simulate the dynamic behaviour of this biochemical network, the kinetic equations were associated with each reaction.

**Conclusions:**

Our model simulates the mechanism of the inactivation of the PBP by methicillin, as well as the expression of PBP2a isoform, the regulation of the SCCmec elements (SCC: staphylococcal cassette chromosome) and the synthesis of peptidoglycan by PBP2a. The obtained results by our integrated approach show that the model describes correctly the whole phenomenon of the methicillin resistance and is able to respond to the external perturbations in the same way of the real cell. Therefore, this model can be useful to develop new therapeutic approaches for the methicillin control and to understand the general mechanism regarding the cellular resistance to some antibiotics.

## Introduction

Microorganisms are able to preserve their functional features against external perturbations. This represents often the major impediment to discover an efficient pharmacological therapy against human pathogens. In fact, the target of a drug is often a specific molecular component of the microorganisms that are easily able to develop new molecular mechanisms for neutralizing the drug presence [1]. For example, it has been recently solved the structure of a protein that adds a methyl group to ribosomal RNA and confers antibiotic resistance to bacteria [2]. Therefore, it has been defined as “robustness” the ability of the living systems to oppose to external perturbations or fluctuations to preserve some critical functional characteristics [3].

It is now known that the functional elements of a cell, i.e. genes, RNAs, proteins, metabolites, etc, establish an integrated network being at the basis of the regulation of cellular biochemical pathways. This network exerts dynamically the cellular robustness against any external perturbation for minimizing its effects on the whole biochemical behaviour of the system. Each functional element has to be considered as a node of a network that carries weighed connections with the other metabolic nodes [4]. At present, we often know in details all molecular properties of each biochemical element as well as the biochemical relationships existing within each “omic” level but we still have poor knowledge of the biochemical laws that connect the various “omic” levels. This represents an important aspect to understand how the biological information is transmitted within the various functional levels as well as to clarify how the cell regulates its functional processes and how we can act on them. Some efforts have been made to model in mathematical terms (by graphs, neural networks, etc.) the relationships among nodes of the same biochemical environment, i.e., relationships among genes at genomic level, among proteins at proteomic level, among mRNA at transcriptomic level, and so on, but few efforts have been made to integrate the information in vertical sense, i.e., among the different functional levels [5].

Lacking this important integration, very hardly we will be able to understand and to model the complex biochemical behavior as a whole [3], [6], because its biological response is produced trough the integration between the different biochemical functional levels.

The antibiotic resistance, developed by different bacteria strains, is a clear example of robustness and of ability of the bacterial system to acquire a particular functional behaviour in response to environmental changes [7]. The molecular mechanisms, related to each functional level of the bacterial cell against some antibiotics are known and even moderately simple. Therefore, we have considered this phenomenon for a modelling of the integrated cellular response to an external perturbation. The modelling reflects the integrated response of the bacterial cell that through its robustness is able to cause the “emergence” of new biological properties.

The first form of drug resistance was the so called “penicillin-resistance”, due to penicillin, an antibiotic of the beta lactamic family. Its resistance is due to the ability of bacteria to express beta-lactamase, an enzyme that can inactivate the drug by hydrolysis of its beta lactamic ring [8], [9]. To overcome this phenomenon, it was necessary to develop new drugs not affected by the same molecular mechanisms of inactivation. One of the defining features of bacterial resistance is based on the physical properties of cell wall representing possible barriers for attacks by pathogens.

**Figure 1:**
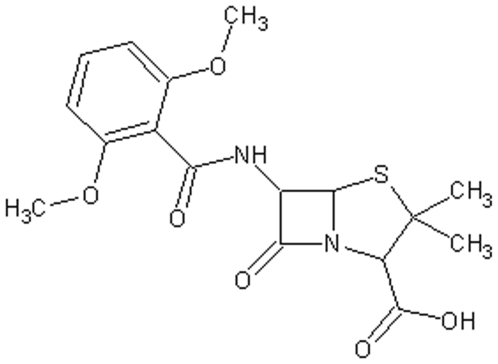
The methicillin structure.

The methicillin being a new synthesized antibiotic (see Figure 1) was checked against the penicillin-resistant strains with excellent results because it is not a beta-lactamase substrate, although the methicillin target is the same of the penicillin. In fact, both drugs are β-lactamic antibiotics that act by inhibiting the penicillin-binding proteins (PBPs). PBPs are involved in the synthesis of peptidoglycans, essential mesh-like polymers that surround cellular enzymes and are crucial for the bacterium survival.

Once the methicillin was clinically used, the patients developed methicillin-resistant strains, as for example the methicillin-resistant *S. aureus* (MRSA) strains. For these reasons, the functional use of this drug is now reduced to few cases [10], [11]. The methicillin-PBPs interaction causes the inactivation of the normal path to synthesize the peptidoglycan. In MRSA strains the resistance mechanism is based on the PBP2a expression. Moreover, some genes (mecI_GENE, mecA_GENE and mecR1_GENE) are located on the mobile genetic elements, known as SCCmec elements. In particular, the mecA_GENE encodes for PBP2a when the methicillin arrives, mecR1_GENE for membrane-bound signal transduction protein (mecR1_PROTEIN) and mecI_GENE for a transcriptional regulator. These PBP2a isoforms present a decrease of binding affinities for antibiotics. Therefore, PBP2a confers resistance by contributing to the function of native PBPs during cell wall synthesis [10], [11].

Rates of invasive infections with methicillin-resistant *S. aureus* (MRSA) have increased both in the hospital and in the community. The prevalence of methicillin-resistant S. aureus (MRSA) worldwide [12] is continuously increasing. This spread of virulent community associated MRSA [13] is accompanied with the emergence *S. aureus* and a reduced susceptibility to other antibiotics, such as vancomycin and other glycopeptides [13], [14], [15]. Vancomycin, that is the antibiotic used for MRSA infections for the past 40 years, does not seem to be as effective [16] due to the appearance of vancomycin-resistant *S. aureus* following the emergence of vancomycin-resistant enterococcus [17]. Moreover, it was experimentally reported that benign-appearing skin and soft tissue infections caused by MRSA can progress rapidly to potentially fatal diseases [18].

What is really worrying are the recent news which report of patients that have been infected with strains of *S. aureus* resistant to methicillin and vancomycin having also acquired the ability to release cellular toxins [19]. This is raising the spectre of the worst kind of antibiotic-resistant superbug [19]. Studies on *S. aureus* conducted for determining the minimum inhibitory concentration at microbial population level have revealed the complex response to drug exposure [20].

Therefore, it is necessary to understand at metabolic level the molecular dynamics that support the persistent *S. aureus* bacteremia. Several research groups have developed strategies for the control and the treatment of MRSA infections through modeling approaches. Brandner et al. offer a platform to undertake high-throughput genomic and proteomic studies of *S. aureus* and MRSA infection, including the molecular mechanisms involved in transmission, virulence, immune-escape, and antibiotic resistance. They reported the results of the entire cloning set of *S. aureus* protein-encoding open reading frames (ORFs), or ORFeome [21]. Moreover, a recent work focus on the importance of different kinetic parameters associated with the resistance mechanisms, using a computational approach. Murphy et al. created a model that can be used to generate quantitatively accurate predictions of MICs for antibiotics against different strains of MRSA and to quantify the effects of the principal pharmacokinetic parameters of these antibiotics on treatment, investigating the relative impact of to β-lactam antibiotics on cell survival in the presence of antibiotics [22].

We have integrated cooperatively the different functional parts of the bacterial cell in order to exert drug-resistance. In particular, we have integrated the molecular information existing at the various functional levels (genomics, transcriptomics, proteomics, metabolomics) to model the whole mechanism essential to the methicillin-resistance through a systems biology approach that shows the behaviour of a single bacterial cell. In this context, a systems biology approach can be useful to provide a framework, at cellular level, able to define the timing as well as the hierarchy of the metabolic reactions of drug resistance of *S.aureus* that is missing in the literature.

## Results

### Description of the model

**Figure 2:**
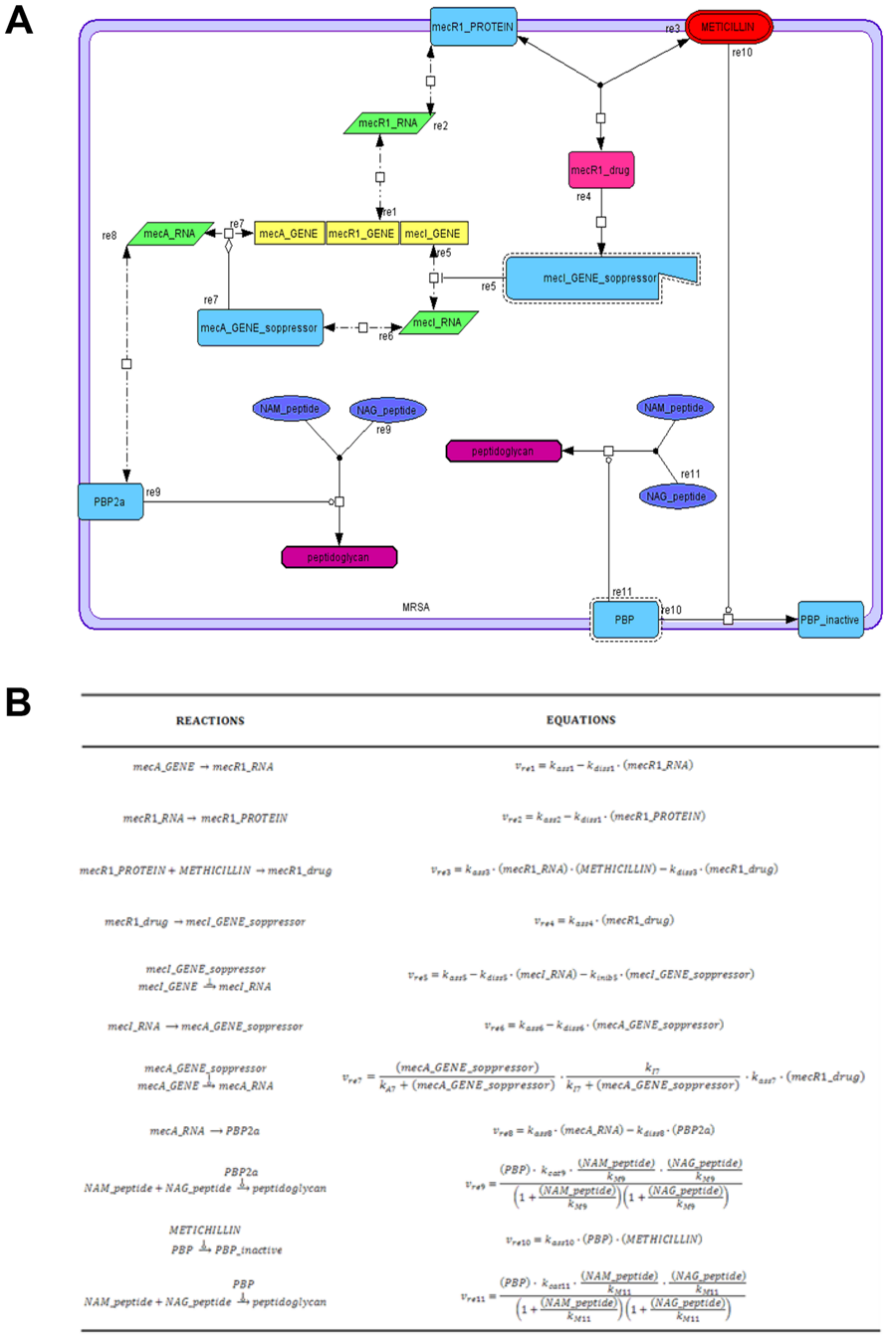
A) Map model. Representation of the network connecting genes, RNAs, proteins and metabolites by using different forms and colours for some species. The frame in light violet represents the cellular membrane. B) Reactions and rate of the equations used in the model.

We created a model to simulate the mechanism of inactivation of the PBP by methicillin, as well as the expression of PBP2a, the regulation of the SCCmec elements (SCC: staphylococcal cassette chromosome) [23], and the synthesis of peptidoglycan by PBP2a. The model and the reaction equations for the model are shown in Figure 2.

The peptidoglycan is obtained from the transpeptidation of NAM and NAG groups (N-acetylmuramic acid and N-acetylglucosamine repeats). In particular, the methicillin-resistance is not due to the production of the β-lactamase enzyme, as one can see in the case of the penicillin-resistance, but depends on the expression of a penicillin-binding protein (PBP2a). This foreign protein is resistant to the action of the methicillin, as demonstrated by the low affinity of PBP2a for the methicillin (METHICILLIN) [8], [9]. In the chromosome of methicillin-resistant strains was found the mecA_GENE, that express PBP2a, and a series of other genes located on mobile genetic elements, known as SCCmec elements. The mecR1_GENE encodes a membrane-bound signal transduction protein (mecR1_PROTEIN) while mecI_GENE encodes a transcriptional regulator (mecA_GENE_repressor), which is a strong repressor of the expression of the mecA gene. When MecR1 protein interacts with the methicillin, it breaks off and its fragment (mecI_GENE_soppressor) is able to repress the mecI_GENE. So, in the absence of the mecA_GENE_soppressor, the mecA_GENE can express PBP2a. The PBP2a expression enables the cell to synthesize the peptidoglycan and this neutralizes the methicillin effect.

In the proposed model 11 reactions are involved. In particular, Re1, Re2, Re6, Re7 and Re8, are defined as zeroth order forward, first order reverse, reversible mass action kinetics; Re3 as second order forward with two reactants, first order reverse, reversible mass action kinetics (generalized mass-action); Re4 as first order irreversible mass action kinetics; Re10 as first order irreversible mass action kinetics (catalyzed by METHICILLIN); and Re9 and Re11 as irreversible non modulated non-interacting bireactant enzymes. (Appendix A, Table S1 and Table S2 in Supplementary Material). Moreover, we modified the equation of the reaction Re7 concerning the complex meticillin-mecRI concentration as our variable, and we included in the equation of the reaction Re5 mecI_GENE_soppressor as our inhibitory variable.

The model presents the framework of genes, RNAs, enzymes, products and reactions involved in the methicillin-resistence phenomenon. In particular, it comprises 6 proteins, 3 genes, 3 RNAs, 1 drug, 2 generic-Molecule, 1 complex and 1 interaction entity (Supplementary Table S2) and the following reactions: 2 state transitions, one of this catalysed by the meticillin drug, 3 associations, (2 of this catalysed by PBP and PBP2a enzymes), 2 transcriptional controls, 3 transcriptions and 3 traslations (Supplementary Table S1 and Table S2).

### Model simulations

This paper describes the results of an integrated approach to model the phenomenon of resistance to methicillin in *S. aureus* at molecular level. This should provide a basis for understanding the dynamics involved in the cellular development of methicillin resistance that can be able to develop pharmacological strategies to overcome its resistance.

We have not used real concentrations for every molecular specie because there are no experimental investigations that reported quantitative data for a single cell. Nevertheless, literature studies [23]–[25] follow us to determinate the relationships between the species in the model and the framework between them was established. We used for the constants (association, dissociation, catalytic and inhibition) the values ranged between 0.1 and 2 (see Supplementary Table S1) concerning the following biological considerations. In details, the catalytic constant (kcat) may be higher than that of association (kass) because it has a role speeding the reaction but the association constant (kass) may be higher than those of dissociation (kdiss) and inhibition (kini) having only a regulatory and control role in the reaction in which these are involved. In fact, when we used values out from 0.1<k<2 or from the criteria reported above, the simulation produced wrong results: i) negative concentrations for species if equal values were set for all constants or if kass>kcat; ii) the meticillin effect was minimal if kass<kdiss and iii) the peptidoglycan increase was slow if kcat<kdiss and kcat<kass (Supplementary Figure S1).

All the molecular species are considered as variable species, except the genes that are assumed constants because in a cell a gene is always present, even if not expressed. When the transcription is activated, the RNA is expressed and the variable of the transcription process is the transcript amount, and not the presence of the gene that can be activated or inactivated.

In absence of quantitative data regarding a single cell, we can only assess the internal ratios existing among reactants or their fluxes. Therefore, we refer to these in terms of amounts, to evidence their presence or their absence. In particular, for the gene class we can not define a concentration, assuming the gene as a constant, and we set every one equal to an unit of amount. Some amounts are fixed at zero because some species, as mecR1_drug, mecI_GENE_soppressor, PBP2a, PBP_inactive, mecA_RNA, initially are not expressed. MecR1_RNA is set equal to 1 as well as the corresponding mec_R1_protein because these values represent basal amounts to ensure the activation of the gene and its relative expression. Instead, the peptidoglycan, PBP, NAM_peptide and NAG_peptide are initially set equal to 3 as amount because in a synthesis process these species are more active respect to mec_R1_protein being like a “sentinel” protein that attends only when the methicillin arrives. MecA_GENE_soppressor and mecI_RNA are set equal to 5 as amount. This is the highest value because it represents the more important transcriptional control of the bacterial resistance involved in the simulation. When PBP enzymes are inactivated by the methicillin, PBP2a is expressed in order to restart the synthesis of the peptidoglycan. In these conditions it is observed a growing amount of various molecular species involved in the mechanism of resistance, largely based on the expression of PBP2a.

**Figure 3:**
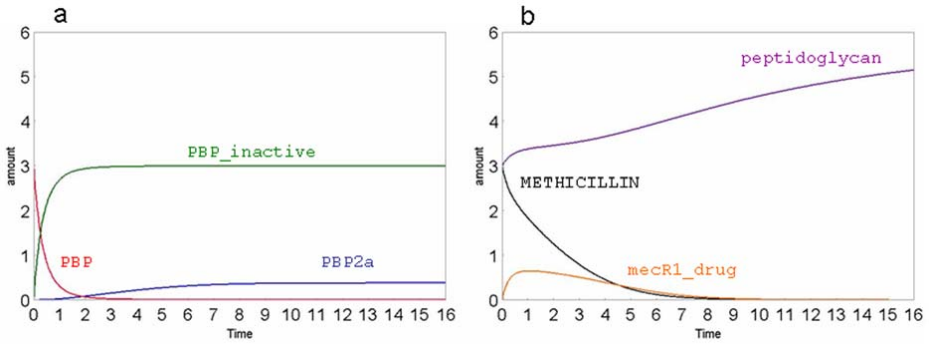
Simulation examples of our model. Flux curves obtained for PBP, PBP-inactive and PBP2a (a), peptidoglycan, METHICILLIN, mecR1_drug (b). The substance amount and time are expressed in number of molecules and seconds, respectively.

In the Figure 3a the variations of PBP, PBP-inactive and the PBP2a are shown. The effect of the methicillin presence is evident because PBP quickly decreases in about one unit of the simulation time and becomes inactive (i.e. PBP-inactive), while the PBP2a is slowly expressed in about seven units of the time scale. The Figure 3b shows that 1) the methicillin amount decreases when it interacts with the mecR1_PROTEIN and inactivates the PBP; 2) the synthesis of peptidoglycan ends when PBPs are inactivated while increases with the espression of PBP2a; 3) the amount of mecR1_drug increases while the methicillin is consumed. It is worthy of note that the total amount of mecR1_drug, spanning from 0 to 6 time units, is about the 25–30% of the total methicillin. This evaluation originates from the analysis of the graphs (see Figure 3b) and is a consequence of the used parameters that were not set on this criteria. In fact, as reported below, some initial parameters were modified in order to verify how the curves change and if the model reply in agreement to cellular mechanism. However, this means that the methicillin affinity for its cellular receptor (mecR) should be not very high.

This is in agreement to a recent article that has found the kinetic data for the binding of penicillin-BlaR to be smaller in comparison to other drugs [24]. Our simulations show that, while in the initial phase of the methicillin action the PBPs are quickly inactivated, the bacterial peptidoglycan growth are consistently reduced (about 55%) whereas the cell wall defence is quickly at the maximum in the absence of methicillin.

In general, these results are consistent with the behaviour of the methicillin-resistant *S. aureus* (MRSA) strains in presence of the antibiotic methicillin but the model highlights new interesting metabolic features of the cell reply. In particular, the whole phenomenon is completely dependent from “de novo” biosynthesis of peptidoglycan. The normal path to synthesize this structure is rapidly inactivated by methicillin (see Figure 2). The new path, i.e. the cellular response, occurs through the expression of PBP2a. Therefore, the computational model shows that these two mechanisms have different kinetics. As shown in Figure 3b, the inactivation of the normal path is immediate with the entrance of methicillin, because it happens simply through the PBP inactivation. The cell reply depends on the mecR1_drug increase that is related to the interaction between methicillin and mecR1_protein, and is not directly linked to the total amount of methicillin, being also used to inactivate PBP. This reply is also correlated to PBP2a expression that needs a complete transcription, translation and expression process. Therefore, the cellular response of defence is slow compared to the action of the methicillin.

**Figure 4:**
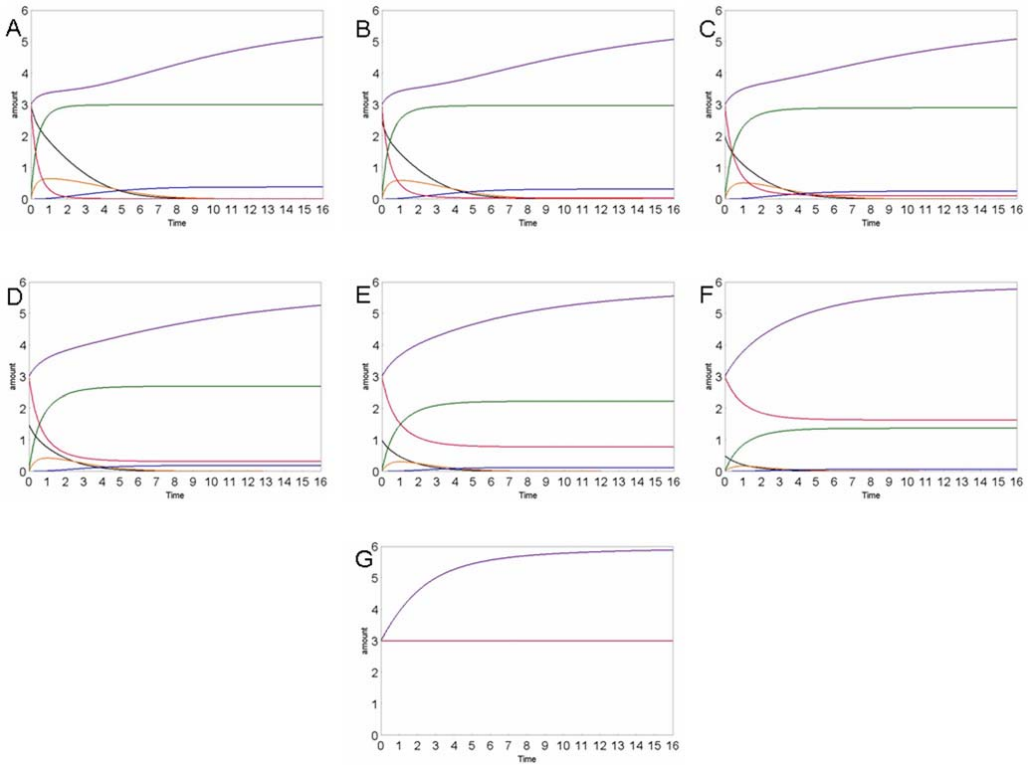
Concentration curves of the various species: methicillin (black), peptidoglycan (violet), PBP (red), PBP2a (dark blue), PBP_inactive (green), mecR1_drug (orange), in different simulations, using different METHICILLIN amounts: A: 3; B: 2.5; C: 2; D: 1,5; E: 1; F: 0,5; G: 0. The various species are distinguished by colours as indicated in the legend. The substance amount and time are expressed in number of molecules and seconds, respectively.

**Figure 5:**
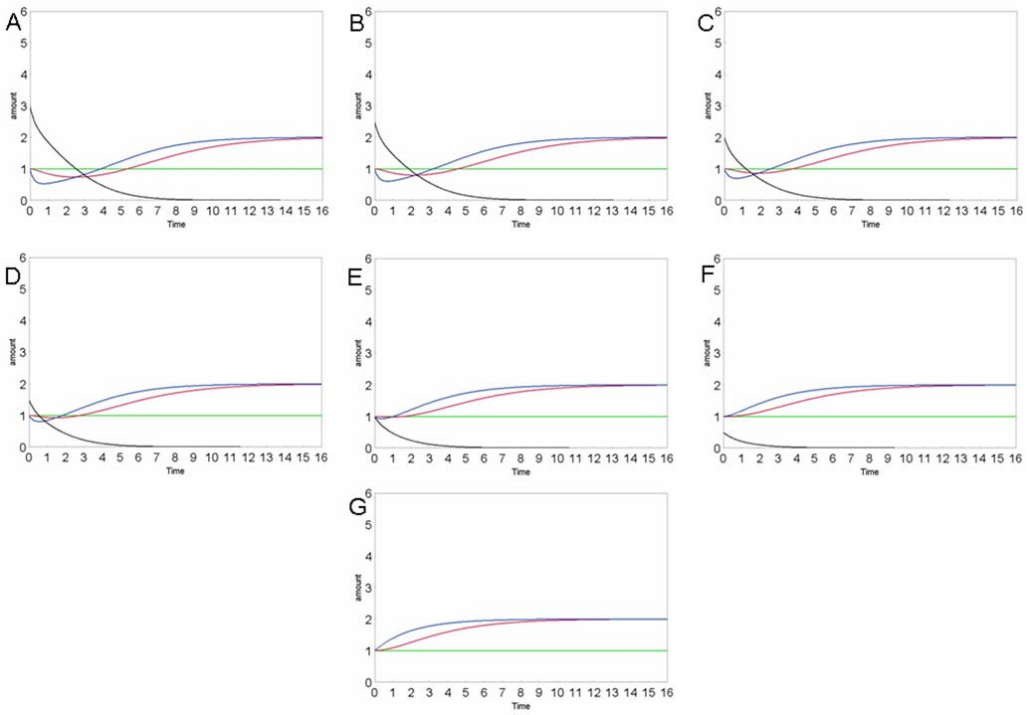
Concentration curves of the various species: methicillin (black), all genes (green), mecR1_protein (blue), mecR1_RNA (red), in different simulations, using different METHICILLIN amounts: A: 3; B: 2.5; C: 2; D: 1,5; E: 1; F: 0,5; G: 0. The various species are distinguished by colours as indicated in the legend. The substance amount and time are expressed in number of molecules and seconds, respectively.

We have also perturbed our model with different methicillin amounts to verify if it responds correctly to perturbations. In fact, as one can easily control, the different concentrations of methicillin modulate the cellular expression of PBP2a in proportion as well as the peptidoglycan synthesis. In Figure 4 and Figure 5, we show the results of different simulations.

In particular, the final amount of peptidoglycan is the same in all conditions, being dictated by the need of the cell to synthesize the wall protection. This agrees to the fact that the penicillin can heavily attack the bacteria if they are in a growth stasis but if the bacteria have lost their cell wall, they can grow and replicate in presence of the antibiotic (L forms) that doesn't appear toxic [25].

The kinetics of its synthesis depend on the presence of PBP and PBP2a enzymes, and are strongly influenced by the amount of methicillin, that induces their inactivation and expression, as we shown in the Figure 4.

A large quantity of methicillin causes a rapid inactivation of the PBP [9]. This induces a temporary stop of the peptidoglycan synthesis that can be resumed only when the PBP2a expression, being more slow respect to inactivation process, is completed. Moreover, this result reveals an important period of latency that can be used for new pharmacological approach. When the effect of methicillin is poor, the process of inactivation is slower, and the growth curve of peptidoglycan amount is not described by an immediate stop but by a gradual decrease, that is followed by a more rapid recovery of the growth, as shown in Figure 4. The strong correlation between the amount of methicillin in the cell and the rate of the phenomenon has been demonstrated experimentally in infection models by the shorter, or absent, post-antibiotic effects [26].

**Figure 6:**
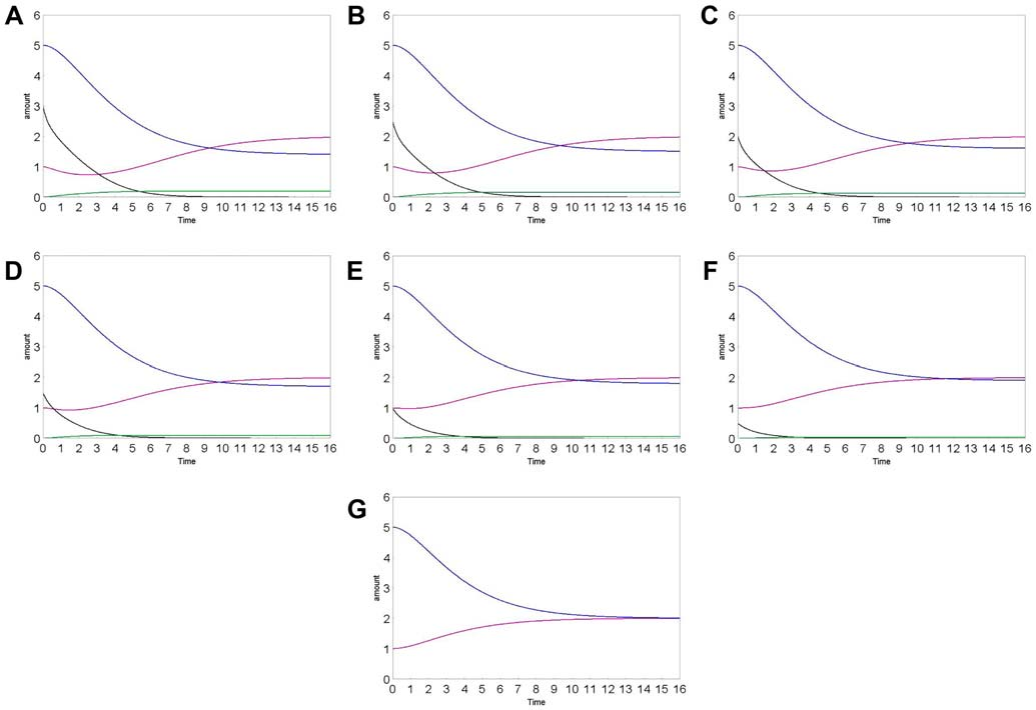
Concentration curves of the various species: methicillin (black), mecA_RNA (green), mecR1_RNA (purple), mecI_RNA (blue) in different simulations, using different METHICILLIN amounts: A: 3; B: 2.5; C: 2; D: 1,5; E: 1; F: 0,5; G: 0. The various species are distinguished by colours as indicated in the legend. The substance amount and time are expressed in number of molecules and seconds, respectively.

Furthermore, we focused our attention on the expression of mecR1, in terms of gene, RNA and protein (Figure 5). The protein mecR1 is the first involved in the detection of methicillin. This protein activates the response of the cell after the drug binding. Our simulation shows that, when the perturbation is due to a small amount of methicillin, the cell is able to cover up quickly the right amount of protein mecR1 with its concomitant transcription. Instead, when the cell perturbation is due to a great amount of methicillin, the transcription process to express mecR1 is not able to immediately satisfy its consumption. This highlights the correlation between the efficacy of the treatment and the doses used. Moreover, we have also investigated the fluxes of all RNA species. The simulations with different amount of methicillin (Figure 6) show that the behaviour of RNA curves agrees with the biological expectations. In fact, in the absence of methicillin, mecA_RNA is not expressed since it is not necessary to express PBP2a, but when methicillin is assumed, both PBP2a and its related RNA are expressed. As mentioned before (Figure 5), mecR1_RNA shows different kinetics at different methicillin amounts because mecR1_protein is consumed and, consequently, the corresponding RNA fluxes must change. In fact, mecR1_RNA is expressed only in the absence of methicillin and it is consumed to inhibit PBP2a expression. However, its amount can't fall below a certain level to maintain the inhibition. We have also verified that, with the presence of methicillin, mecI_RNA falls below this threshold to stop the inhibition as well as to allow the PBP2a expression.

## Discussion

Our integrated approach shows that a computational model can generate and describe correctly the whole phenomenon of the methicillin resistance at biochemical level. The concept of integrated biological system has emerged as a means of envisioning how multifactorial biological processes operate as a whole. Here, we attempted to evaluate the current state of knowledge about the peptidoglycan components of MRSA wall in the context of methicillin action. The model suggests that two metabolic cascades, both activated by the methicillin, display strikingly different levels of temporal response to varying strengths of perturbations. In fact, the expression of the target gene (mecI_gene) in the metabolic cascade was influenced by the upstream reactions. This finding suggests that network connectivity has a greater effect on the variability of expression than the expression of a gene itself. The model is able to accurately predict the latency time of single genes within the two cascades demonstrating that such behaviour can be described in a quantitative manner.

Experimentally it's known that, when the methicillin is assumed by patients, PBP is inactivated but PBP2a is expressed and the peptidoglycan synthesis is induced [8], [9]. According to these data the curves reported in Figure 3 showed that after methicillin administration the PBP amount decreased while the PBP2a and peptidoglycan amounts increased. Moreover, the model is also able to react to the external perturbation (i.e. to different amounts of methicillin) by activating the same biochemical mechanisms of the *S. aureus* and modulating its response on the basis of the extent of the perturbation. In fact, when the model is perturbed with different amounts of methicillin, its reply is proportional to the perturbation. The strong correlation between the amount of methicillin in the cell and the rate of the phenomenon has also been observed by the very short, or missing, post-antibiotic effects (PAE) in experimental models of infection [26]. The “post-antibiotic effect” term refers to the time period after complete removal of an antibiotic during which there is no growth of the target organism. This is a feature of most antibiotics agents influenced by several factors, such as organism type, antibiotic type, and treatment type. The beta-lactamic class has demonstrated both *in vivo* and *in vitro* a post-antibiotic effect against gram-positive *cocci* but not against gram-negative *bacilli* [26], [27] because of the structural complexity of the cell-wall of negative bacteria which is opposed to the entry of the methicillin in the cytoplasmic space. For this reason, the beta-lactamic treatment requires frequent or continuous dosing.

The computational results demonstrate that the cellular response depends on the effective amount of methicillin inside the cell. Therefore, the knowledge of the integrated metabolic behaviour can be useful to develop new therapeutic approaches to control the methicillin resistance as well as a useful methodological tool for deepening the understanding of the mechanism on the cellular resistance to antibiotics. In fact, the time period between the end of the path of the PBP inactivation and the activation of the second path of the PBP2a expression corresponds to a latency of the peptidoglycan biosynthesis. Visibly this is a period in which the bacterium might be vulnerable. Moreover, the correlation between the methicillin amount and the expression rate of some proteins (see mecR1 in Figure 5) should also support a detailed study of therapeutic doses.

In conclusion, our results show that the integrated approach of the systems biology, that we have applied for studying the mechanism of the bacterial resistance to methicillin at biochemical level, can be very useful to a biological modelling of complex infectious diseases.

## Methods

To develop models of integrated biochemical levels, it is necessary to consider the mechanisms by which biochemical information transfer occurs. The network of genes, mRNA, proteins and metabolites was created using CellDesigner version 4.0 (http://celldesigner.org/), a software that enables users to describe molecular interactions using a well-defined and consistent graphical notation [28]. The data of molecular interactions are stored in Systems Biology Markup Language (SBML; http://sbml.org/) [29]. Since SBML is a standard machine-readable model representation format, all the information can be used for a range of computational analysis, including computer simulation [30].

To simulate the dynamic behavior of these biochemical networks, kinetic equations have to be associated with each reaction. The software SBMLsqueezer was used to generate kinetic rate equations for our biochemical network. This approach facilitates the modeling steps via automated generation of equation and overcomes the highly error-prone and cumbersome process of manually assigning kinetic equations. For each reaction the kinetic equation is derived from the stoichiometry, the participating species (e.g., proteins, mRNA or generic molecules) as well as the regulatory relations (activation, inhibition or other modulations) of the SBGN diagram. The software SBMLsqueezer offers different types of kinetics (i.e. mass-action, Hill, and several Michaelis-Menten-based kinetics), each including activation, inhibition and reversibility or irreversibility for representing gene regulatory, signal transduction, protein, metabolic, and mixed networks. The rate laws were generated by considering for each reaction all participating reactants, products and regulators. In particular, for gene regulatory networks, i.e., transcriptional and translational processes, the Hill equation is applied.

After invoking SBMLsqueezer, the kinetic formulas are generated and assigned to the model, which was then simulated in CellDesigner ver 4.0. Details on reaction type and differential equations are reported in Figure 2B, Supplementary Appendix S1, Table S1 and Table S2 and Supplementary Figure S2. Moreover, the substance amount and time are expressed in number of molecules and seconds, respectively (see for details Table 3 in Hucka et al (2003) [31]).

## Supporting Information

Appendix S1 Differential's equations used in the model.
(0.18 MB DOC)

Figure S1 Simulation results with different parameters: a) the constants are fixed as indicated for the final model, b) all the constants are set equal to 1, c) all the dissociation constants are set equal to 4, being higher than the association constant, d) all the catalytic constants are set equal to 0.1, being lower than the association constant, e) all the association constants are set equal to 0.1, being lower than the dissociation constant, f) one of the association constants is set equal to 4, being out from the established range. The substance amount and time are expressed in number of molecules and seconds, respectively.
(0.43 MB DOC)

Figure S2 Graphical notations and colors used to represent the network (CellDesigner ver4.0).
(0.04 MB DOC)

Table S1 Details regarding the reactions type and the names used to indicate both reactants and products and the parameters values.
(0.04 MB DOC)

Table S2 Details on initial quantities of all species used during the simulation.
(0.05 MB DOC)
