# Supplementary material for: Modeling of the Bacterial Mechanism of Methicillin-Resistance by a Systems Biology Approach
Source: PLoS One. 2009 Jul 13;4(7):e6226. doi: 10.1371/journal.pone.0006226 (PMC2707609; doi:10.1371/journal.pone.0006226)

**Appendix S1**

For **Re1**, **Re2**, **Re3**, **Re6**, and **Re8** we used several cases of reversible generalized mass-action (i.e. first order reverse, second order forward, zeroth order forward) according to Systems Biology Ontology (SBO).

The related differential equation is:


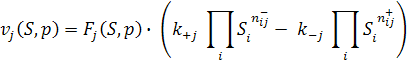


where

S= reacting species

p= parameter vector

k_+j_ = association constant

k_-j_ = dissociation constant

For **Re7** we used a special case of irreversible generalized mass-action (irreversible modulated mass-action) in which Fj(S,p) is equal to fj(S,p) of inhibition law.

The related differential equation is:


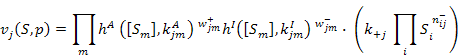


where

S= reacting species

p= parameter vector

k_+j_ = association constant

k^A^= association constant of mecA_GENE_soppressor

k^I^=inhibition constant of mecA_GENE_soppressor

For **Re4** and **R10** we used irreversible generalized mass-action.

The related differential equation is:


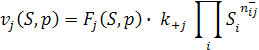


where

S= reacting species

p= parameter vector

k_+j_ = association constant

For **Re9** and **Re11** we used irreversible non-modulated non-interacting reactant enzymes.

The related differential equation is:


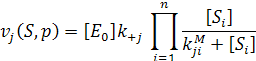


where

S= reacting species

p= parameter vector

k_+j_ = association constant

k^M^ji= catalytic constant

E=enzyme

For **Re5** we used inhibition laws.

The related differential equation is:


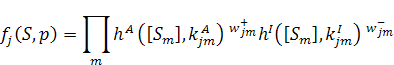


where


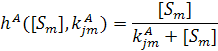


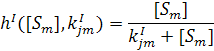

Supplement: Appendix S1 — Differential's equations used in the model. (0.18 MB DOC) [file pone.0006226.s001.doc]
