# Supplementary material for: Modeling of the Bacterial Mechanism of Methicillin-Resistance by a Systems Biology Approach
Source: PLoS One. 2009 Jul 13;4(7):e6226. doi: 10.1371/journal.pone.0006226 (PMC2707609; doi:10.1371/journal.pone.0006226)
Supplement: Figure S1 — Simulation results with different parameters: a) the constants are fixed as indicated for the final model, b) all the constants are set equal to 1, c) all the dissociation constants are set equal to 4, being higher than the association constant, d) all the catalytic constants are set equal to 0.1, being lower than the association constant, e) all the association constants are set equal to 0.1, being lower than the dissociation constant, f) one of the association constants is set equal to 4, being out from the established range. The substance amount and time are expressed in number of molecules and seconds, respectively. (0.43 MB DOC) [file pone.0006226.s002.doc]

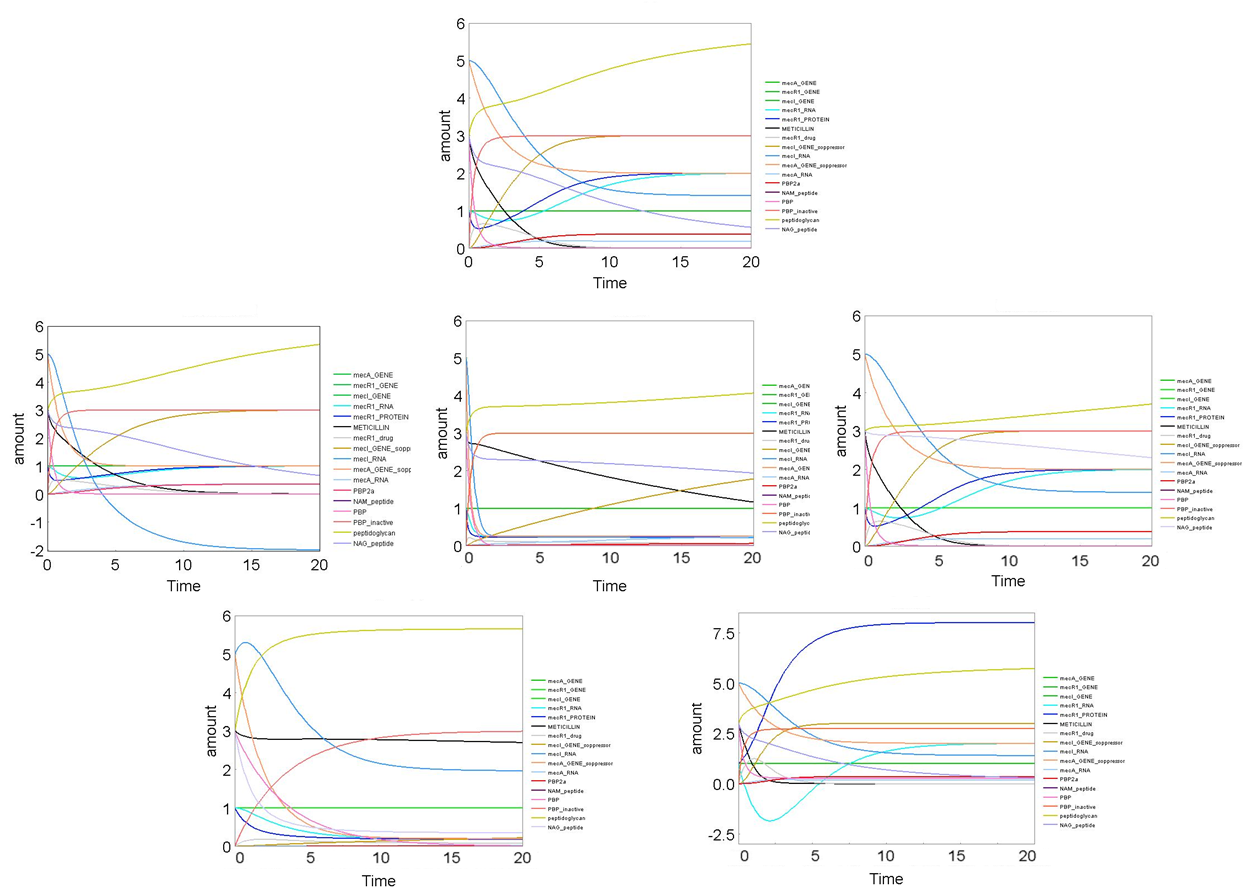


d

e

f

c

b

a

**Figure S1.** Simulation results with different parameters: **a)** the constants are fixed as indicated for the final model, **b)** all the constants are set equal to 1, **c)** all the dissociation constants are set equal to 4, being higher than the association constant, **d)** all the catalytic constants are set equal to 0.1, being lower than the association constant, **e)** all the association constants are set equal to 0.1, being lower than the dissociation constant, **f)** one of the association constants is set equal to 4, being out from the established range. The substance amount and time are expressed in number of molecules and seconds, respectively.
