# Supplementary figures and images for: Modeling of the Bacterial Mechanism of Methicillin-Resistance by a Systems Biology Approach
Source: PLoS One. 2009 Jul 13;4(7):e6226. doi: 10.1371/journal.pone.0006226 (PMC2707609; doi:10.1371/journal.pone.0006226)

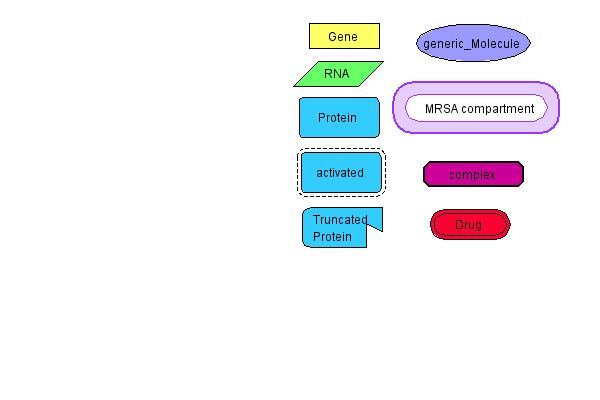


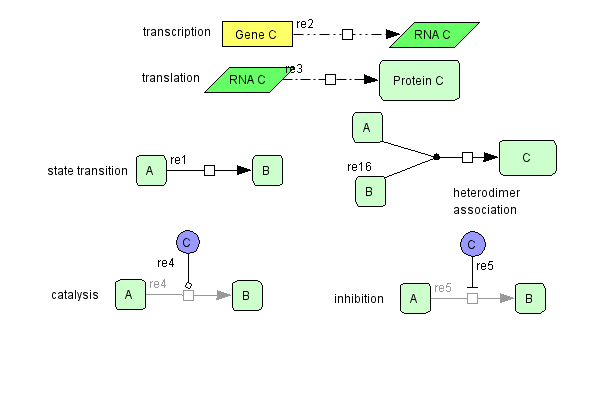


**Figure S2**. Graphical notations and colors used to represent the network (CellDesigner ver4.0).

Supplement: Figure S2 — Graphical notations and colors used to represent the network (CellDesigner ver4.0). (0.04 MB DOC) [file pone.0006226.s003.doc]
