# Supplementary material for: Modeling of the Bacterial Mechanism of Methicillin-Resistance by a Systems Biology Approach
Source: PLoS One. 2009 Jul 13;4(7):e6226. doi: 10.1371/journal.pone.0006226 (PMC2707609; doi:10.1371/journal.pone.0006226)
Supplement: Table S1 — Details regarding the reactions type and the names used to indicate both reactants and products and the parameters values. (0.04 MB DOC) [file pone.0006226.s004.doc]

**Table S1.**

Details regarding the reactions type and the names used to indicate both reactants and products and the paramters values.

| **Reactions** | **TYPE** |  | **k ID** | **value** | **reactants** | **products** | **modifiers** |
| --- | --- | --- | --- | --- | --- | --- | --- |
| **Re1** | TRANSCRIPTION |  | Kass1 Kdiss1 | 1 0,5 | mecA_GENE | mecR1_RNA |  |
| **Re2** | TRANSLATION |  | Kass2 Kdiss2 | 1 0,5 | mecR1_RNA | mecR1_PROTEIN |  |
| **Re3** | HETERODIMER_ASSOCIATION |  | Kass3 Kdiss3 | 1 0,5 | mecR1_PROTEIN METICILLIN | mecR1_drug |  |
| **Re4** | STATE_TRANSITION |  | Kass4 | 1 | mecR1_drug | mecI_GENE_soppressor |  |
| **Re5** | TRANSCRIPTION |  | Kass5 Kdiss5 Kinib5 | 1 0,5 0,1 | mecI_GENE | mecI_RNA | mecI_GENE_soppressor |
| **Re6** | TRANSLATION |  | Kass6 Kdiss6 | 1 0,5 | mecI_RNA | mecA_GENE_soppressor |  |
| **Re7** | TRANSCRIPTION |  | Kass7 KA7 KI7 | 1 1 1 | mecA_GENE | mecA_RNA | mecA_GENE_soppressor |
| **Re8** | TRANSLATION |  | Kass8 Kdiss8 | 1 0,5 | mecA_RNA | PBP2a |  |
| **Re9** | HETERODIMER_ASSOCIATION |  | Kcat9 KM9 KM9 | 2 2 2 | NAM_peptide NAG_peptide | peptidoglycan | PBP2a |
| **Re10** | STATE_TRANSITION |  | Kass10 | 1 | PBP | PBP_inactive | METICILLIN |
| **Re11** | HETERODIMER_ASSOCIATION |  | Kcat11 KM11 KM11 | 2 2 2 | NAM_peptide NAG_peptide | peptidoglycan | PBP |
