# Supplementary material for: Modeling of the Bacterial Mechanism of Methicillin-Resistance by a Systems Biology Approach
Source: PLoS One. 2009 Jul 13;4(7):e6226. doi: 10.1371/journal.pone.0006226 (PMC2707609; doi:10.1371/journal.pone.0006226)
Supplement: Table S2 — Details on initial quantities of all species used during the simulation. (0.05 MB DOC) [file pone.0006226.s005.doc]

**Table S2**

Details on initial quantities of all species used during the simulation.

| **Class** | **Entity** | **Position** | **Quantity type** | **Initial quantity** | **Constants** |
| --- | --- | --- | --- | --- | --- |
| **GENE** | mecA_GENE | inside | Concentration | 1.00 | True |
| **GENE** | mecR1_GENE | inside | Concentration | 1.00 | True |
| **GENE** | mecI_GENE | inside | Concentration | 1.00 | True |
| **RNA** | mecR1_RNA | inside | Amount | 1.00 | False |
| **PROTEIN** | mecR1_PROTEIN | transmembrane | Amount | 1.00 | False |
| **DRUG** | METICILLIN | transmembrane | Amount | 3, 2.5, 2, 1.5, 1, 0.5, 0 | False |
| **PROTEIN** | mecR1_drug | inside | Amount | 0.00 | False |
| **PROTEIN** | mecI_GENE_soppressor | inside | Amount | 0.00 | False |
| **RNA** | mecI_RNA | inside | Amount | 5.00 | False |
| **PROTEIN** | mecA_GENE_soppressor | inside | Amount | 5.00 | False |
| **RNA** | mecA_RNA | inside | Amount | 0.00 | False |
| **PROTEIN** | PBP2a | transmembrane | Amount | 0.00 | False |
| **GENERIC_MOLECULE** | NAM_peptide | inside | Amount | 3.00 | False |
| **GENERIC_MOLECULE** | NAG_peptide | inside | Amount | 3.00 | False |
| **PROTEIN** | PBP | transmembrane | Amount | 3.00 | False |
| **PROTEIN** | PBP_inactive | transmembrane | Amount | 0.00 | False |
| **COMPLEX** | peptidoglycan | inside | Amount | 3.00 | False |
